# Book Review: Stigma, Discrimination, and Living with HIV/AIDS: A Cross-Cultural Perspective

**DOI:** 10.3389/fpubh.2015.00242

**Published:** 2015-10-29

**Authors:** Joyce Addo-Atuah, William Lundmark

**Affiliations:** ^1^Social/Behavioral/Administrative Sciences, Touro College of Pharmacy, New York, NY, USA; ^2^Touro-Harlem Medical Library, Touro College of Pharmacy and Touro College of Osteopathic Medicine, New York, NY, USA

**Keywords:** HIV/AIDS, HIV/AIDS stigma, HIV/AIDS-related discrimination, global research, cross-cultural perspectives, outcomes of HIV/AIDS stigma and discrimination

Stigma, a socially constructed process that categorizes people into “them” and “us” on the basis of race, gender, sexual orientation, and disease, among others, leads to discrimination against the stigmatized individuals or groups who are devalued in comparison to those in the mainstream. HIV/AIDS-related stigma and discrimination are issues of great concern because of their ramifications on access to HIV/AIDS prevention, treatment, care, and support for people living with HIV/AIDS (PLWHA). HIV/AIDS-related stigma and discrimination have been recognized globally and many reports of these have appeared in the biomedical literature. However, “*Stigma, Discrimination and Living with HIV/AIDS: A Cross-Cultural Perspective*,” edited by Pranee Liamputtong with 43 contributors, is the first book, which attempts to collate in one volume, results from empirical research on the subject matter from a cross-cultural and global perspective. Pranee Liamputtong, who holds a personal chair in public health at the School of Public Health at La Trobe University in Melbourne, VIC, Australia, builds on her extensive research work on the cultural and social influences on reproductive health to address HIV/AIDS-related stigma and discrimination from broad and diversified cultural, geographic, and sub-group perspectives.

“*Stigma, Discrimination and Living with HIV/AIDS: A Cross-Cultural Perspective*,” focuses on the lived stigma and discrimination experiences of PLWHA and how they attempt to deal with these in countries, such as Australia, China, Colombia, India, South Africa, Thailand, the United Kingdom, and the United States. Within the context of these country cultures, layered stigma as experienced by PLWHA already stigmatized on account of their gender, race/ethnicity, sexual orientation, and other infections, such as Hepatitis C, are highlighted in the book.

HIV/AIDS stigma arises from society’s perception of the affected individuals’ deviation from socially accepted standards of “normality.” PLWHA are labeled as being “immoral,” “promiscuous,” or “perverted.” They are, therefore, discriminated against in these settings because they are perceived to be “contagious,” and associated with “death.”

The 415 page book “*Stigma, Discrimination and Living with HIV/AIDS: A Cross-Cultural Perspective*,” is divided into 3 parts preceded by an introductory chapter by the editor, which provides a synopsis of each of the 23 chapters of the book. Each of these chapters is written by one or more researchers who have used mainly qualitative research methods to gather real life data of the lived stigma and discrimination experiences of PLWHA in different countries and geographic regions of the world.

Part 1 of the book (chapters 1–6) provides a theoretical basis for understanding HIV/AIDS-related stigma and discrimination from individual, family, and institutional perspectives, and how they interact with other social prejudices, including homophobia, sexism, and racism. Of particular concern and interest here is the stigma and discrimination experienced by PLWHA at the hands of healthcare providers in a variety of healthcare settings. As reported by PLWHA, these take the form of delayed, inadequate, or differential treatment, which directly impacts their health outcomes and quality of life.

Part II (chapters 7–18) addresses the lived HIV/AIDS-related stigma and discrimination experiences across social and cultural settings and beliefs around the globe. This part of the book especially highlights the fact that people who before the advent of the HIV/AIDS epidemic were already socially marginalized, stigmatized, or discriminated against by their societies, have turned out to be those that are bearing the brunt of the global HIV/AIDS pandemic. These groups include women, sex workers, the poor, men who have sex with men (MSM), and intravenous drug users (IDUs). The way in which HIV/AIDS stigma is expressed and experienced by PLWHA has been found to be contextual in nature and therefore varies from place to place and from culture to culture.

Chapters 19–23 of “*Stigma, Discrimination and Living with HIV/AIDS: A Cross-Cultural Perspective*” make up Part 3 of the book. This part of the book describes how PLWHA deal with and attempt to manage stigma and discrimination. Strategies described here include membership of support groups, such as associations of PLWHA.

“*Stigma, Discrimination and Living with HIV/AIDS: A Cross-Cultural Perspective*” would be useful for a wide variety of readers, including healthcare providers taking care of PLWHA, students, and teachers in health professional schools, and also in courses, such as anthropology, sociology, social work, and public health.

The real life data of the lived stigma and discrimination experienced by PLWHA around the globe contained in the book makes it an invaluable source of first-hand information on these issues for HIV/AIDS policy makers and program managers for policy and advocacy purposes. The data also inform and equip PLWHA, enabling them to better cope with stigma and discrimination. In particular, it will assist health workers in community health centers and hospitals in understanding issues related to HIV/AIDS and allow them to provide culturally appropriate health care to PLWHA from different social and cultural backgrounds. The book is useful for anyone who is interested in HIV/AIDS-related stigma and discrimination in diverse social and cultural settings.

“*Stigma, Discrimination and Living with HIV/AIDS: A Cross-Cultural Perspective*,” edited by Pranee Liamputtong and published by Springer, is an easy to read text, which ensures that not only those in the social and health sciences can easily read and understand the text. Terminology used is well-defined throughout the book, which is also thoroughly referenced. Chapter summaries and suggestions for further research complete each chapter. The book does not need to be read cover-to-cover; the systematic division of the text into parts, chapters, sub-chapters, and descriptive headings allow the reader to quickly locate required information from any part of the book.

## Author Contributions

JA-A identified this book as occupying a unique position in the available literature on HIV/AIDS-related stigma and hence worthy of review. She read the book and crafted out the structure for the review manuscript. WL read the book and crafted the initial draft which was updated by JA-A.

## Conflict of Interest Statement

The authors declare that the research was conducted in the absence of any commercial or financial relationships that could be construed as a potential conflict of interest.
